# Weight Gain, Schizophrenia and Antipsychotics: New Findings from Animal Model and Pharmacogenomic Studies

**DOI:** 10.1155/2011/459284

**Published:** 2010-12-06

**Authors:** Fabio Panariello, Vincenzo De Luca, Andrea de Bartolomeis

**Affiliations:** ^1^Department of Psychiatry, Centre for Addiction and Mental Health, University of Toronto, 250 College Street, Room 30, Toronto, ON, Canada M5T 1R8; ^2^Dipartimento di Neuroscienze, Sezione di Psichiatria, Laboratorio di Psichiatria Molecolare, University of Napoli “Federico II”, Via Pansini 5, 80131 Napoli, Italy

## Abstract

Excess body weight is one of the most common physical health problems among patients with schizophrenia that increases the risk for many medical problems, including type 2 diabetes mellitus, coronary heart disease, osteoarthritis, and hypertension, and accounts in part for 20% shorter life expectancy than in general population. Among patients with severe mental illness, obesity can be attributed to an unhealthy lifestyle, personal genetic profile, as well as the effects of psychotropic medications, above all antipsychotic drugs. Novel “atypical” antipsychotic drugs represent a substantial improvement on older “typical” drugs. However, clinical experience has shown that some, but not all, of these drugs can induce substantial weight gain. Animal models of antipsychotic-related weight gain and animal transgenic models of knockout or overexpressed genes of antipsychotic receptors have been largely evaluated by scientific community for changes in obesity-related gene expression or phenotypes. Moreover, pharmacogenomic approaches have allowed to detect more than 300 possible candidate genes for antipsychotics-induced body weight gain. In this paper, we summarize current thinking on: (1) the role of polymorphisms in several candidate genes, (2) the possible roles of various neurotransmitters and neuropeptides in this adverse drug reaction, and (3) the state of development of animal models in this matter. We also outline major areas for future research.

## 1. Introduction

Schizophrenia is a chronic disabling mental illness that affects millions of people worldwide [1]. People with schizophrenia suffer increased rates of multiple medical problems [2–5]. Among these issues, weight gain is a common problem, and some epidemiologic data suggest that patients with mental illnesses are at greater risk of developing obesity estimated to be about 2 times more common in patients with schizophrenia (especially women) than the general population [6]. This is due to their life style inherent neglect of personal care (smoking behavior, high-fat diet), barriers to treatment of physical illness, side effects profile of antipsychotic medications, and genetic influences. Thus, it represents a notable example of genetic/environment interaction, but the extent of the involvement of each factor is unclear. Antipsychotic medications have been associated with considerable weight gain, but the mechanisms by which they increase weight and produce metabolic disturbances are not known [7–10]. Normal subjects have a BMI between 18.5 and 24.9 kg/m^2^. Subjects with a BMI from 25 to 29.9 kg/m^2^ are classified as overweight and from 30 to 39.9 kg/m^2^ as obese. Patients with a BMI above 40 kg/m^2^ are classified as extremely obese [11]. The importance of this side effect clearly arises from the following considerations: (1) a significant increase in weight gain may affect the compliance to pharmacotherapy, because it may add to the schizophrenia stigma the stigma of obesity and be indirectly responsible for psychosis relapses; (2) weight gain may increase the risk for diabetes type II; (3) weight gain can be associated with the metabolic syndrome, a very severe condition that has serious implications for overall health and survival due to an increased risk for cardiovascular and malignant disorders. Although clozapine and olanzapine are the second generation antipsychotics (SGAs) most frequently associated with weight gain, all of the antipsychotic agents seem to share this effect as a group to varying degrees that may be because each drug exerts different degree of action on the serotonergic, dopaminergic, cholinergic, histaminergic, and other neurotransmitter systems [12]. Several lines of evidence suggest that aripiprazole and ziprasidone are associated with the lowest weight gain risk [7]. SGAs have been considered for a very long time to be related to tremendous weight gain compared with first-generation antipsychotics (FGAs). Nevertheless, some studies have shown similar weight gain between these 2 groups of treatment. Among first-generation neuroleptics, low-potency fenotiazines may have grater liability for weight gain than butyrrophenones. Molindone and pimozide have been associated with loss of weight in short-term studies, even if the available data are in some way contrasting [8]. The significance of weight gain as relevant side effect of antipsychotics treatment, that can cause the lack of adherence in drug assumption, has been recently discussed by the authors of the Catie study. The rate of discontinuation due to weight gain or metabolic changes was more than twice as great for olanzapine as for the other study medications (9% versus 1% to 4%) [13]. Olanzapine caused significantly more weight gain than any of the other medicines, even after adjustment for duration of treatment [13]. Despite the growing literature on the clinical impact of weight gain and diabetes in schizophrenia during antipsychotics therapy, and the increasing number of pharmacogenetics studies targeting SNPs (single-nucleotide polymorphisms) [14–17] of candidate genes, few reports are focused, at preclinical level, on the potential molecular mechanisms that may be responsible or substantially contribute or can be correlated robustly to this crucial health issue in schizophrenia treatment. It should be recognized that a major problem in preclinical studies is the difficulty in inducing weight gain reliably in animal paradigm of chronic antipsychotics administration [18]. The major problems in that regard are that female, but not male, rodents appear to be sensitive to the weight-promoting effects of antipsychotics. In fact, after chronic antipsychotic treatments, female mice and rats [19–25] increase their weight. On the contrary in male rats, any weight changes are not observed [24, 26, 27]. Besides, weight gain in female rats seems to result from hyperphagia, while males do not change their food intake [19, 27]. It is a hypothesis that these differences in weight and food intake in female rats from males were due to different expressed hormones [28, 29]. These observations do not match the clinical situation in humans in which there are no differences in antipsychotics-induced weight gain and food intake in both males and females [8, 29]. It is even more surprising that compared to the major side effects of first-generation antipsychotics, that is, extrapiramidal movements and specially parkinsonism, no specific neurotransmitter, receptor, or transduction system has been significantly tracked down as the major responsible factor of antipsychotics-induced increase in weight, even after more than twenty years since the introduction in therapy of the first atypical antipsychotic drug, clozapine. The relationship between the occupancy of some receptors, claimed to be relevant for this side effect, and the real-world clinical evidence is still lacking of a clear demonstration and need to be explored more on both a preclinical and clinical level. However, continuing in the similitude, it needs to be acknowledged that also with the typicals, preferentially dopamine D2 blocking agents, there is an important movement disorder that has not been fully explained yet in molecular terms: tardive dyskinesia. Understanding the mechanism(s) related to the weight gain may require recognizing the following issues.

(1) It is important to underline that the molecular mechanism(s) responsible for weight gain could be unrelated to the molecular mechanisms of the antipsychotic action, but that cannot be completely ruled out that the two mechanisms may partially overlap. The recent discovery of significant interaction between orexin [30], a key molecule in food intake control, and dopamine indicate that the above-mentioned overlap is possible.
(2) Any step of the signal transduction pathway that is initiated by antipsychotic-receptor interaction is, at least potentially, a candidate target of the molecular mechanism responsible for the weight gain.
(3) Transcription factors are the amplifiers and regulators of the signal triggered by receptor activation, and it should be of great interest to explore those transcription factors that are related in the CNS with weight gain mechanism control [31].
(4) The receptor-binding profile of typical and atypical antipsychotics may predict, theoretically, the liability for weight gain; however, the real-world outcome may not confirm in straightforward manner the prediction. On the other side, receptors may be involved in the mechanism of weight gain even if they are not included in the receptor-binding profile of an antipsychotic compound. For instance, olanzapine may indirectly affect certain functions mediated by beta-adrenergic receptors even if has not a significant affinity for any of the beta-receptor subtypes [32].
(5) The dissection between central and peripheral mechanisms responsible of antipsychotics weight gain should also be considered.
(6) It should be acknowledged that the link between weight gain, metabolic syndrome, diabetes type II, and dyslipidemia is a complex matter, and it is conceivable that probably multiple mechanisms should be taken into account and that the weight gain-inducing mechanism may follow [33]. Also, it should be clearly noted that many statements regarding the mechanism of weight gain induced by atypical antipsychotics are surprisingly lacking robust and consistent experimental replication and should be cautiously translated to clinical practice.

## 2. Atypical Antipsychotics Receptor Profile and Weight Gain

Even if any step of the signal transduction pathway activated (or inhibited) by antipsychotics can be regarded as potential target responsible for the weight gain liability of these compounds, the interaction with membrane receptors is, probably, the first, functional relevant, step in their cellular and molecular mechanism that gives the right of clinical properties and profile of side effects. It should be acknowledged that trying to match receptor profile and compare the receptor-binding properties of each compound with weight gain liability is not a straightforward task. Several, probably too many, receptors have been considered putative candidates for the weight gain-inducing effect of atypical antipsychotics: among the others 5HT2cR, 5HT3R *α*2R, H1R, and *β*3R. It should not be forgotten that the involvement of membrane receptor in weight gain antipsychotics liability must include also those receptors that may modulate energy expenditure, and this makes the effort to track down the potential mechanisms even more difficult. Finally, it is a more realistic approach to consider that the weight gain side effects may be the result of the combined action of a certain antipsychotic compound on multiple receptors [34]. Despite these difficulties, the antipsychotics receptor profile may represent the most direct mechanistic connection between antipsychotic molecular effects and antipsychotics-induced weight gain. Typical antipsychotics are universally characterized by their antagonistic effect and high affinity for dopamine receptors, particularly D2 receptors [35]. Dopamine antagonism is believed to be the mechanism of these agents' ability to reduce positive symptoms of schizophrenia as well as their tendency to produce EPS [36]. Atypical antipsychotics, in contrast, are antagonists for both serotonin (5-hydroxytryptamine, 5-HT) and dopamine receptors. In general, atypical agents have an enhanced 5-HT2A/D2 affinity ratio and that helps explain why typical and atypical agents may have different clinical effects [37, 38]. The atypical antipsychotics generally have additional affinities for a variety of neurotransmitter receptor subtypes [39]. This complex pharmacology leads to interactions of varying intensity with numerous serotonergic, dopaminergic, histaminergic, adrenergic, and muscarinic acetylcholine receptors. In addition to having distinct binding profiles, some atypical antipsychotics have a unique effect on neurotransmitter receptor activity [40, 41]. While most parts of antipsychotics are dopamine antagonists that block dopaminergic systems throughout the brain, some newer agents, such as aripiprazole [42] and bifeprunox [43], act as partial dopamine agonists. Certain atypical antipsychotics also act as partial agonists at other sites in addition to dopamine receptor sites. Aripiprazole, ziprasidone, and bifeprunox, for example, each display partial agonism at 5-HT1A [44–46] receptors, and clozapine acts as a partial agonist at M1, M2, and M4 muscarinic receptors and an antagonist of M3 and M5 [47]. Similarly, different findings suggest that N-desmethylclozapine, the major active metabolite of clozapine, acts as a partial agonist at M5, a full agonist at M1, and an antagonist at M3 receptors [47].

## 3. Serotonin Signaling

The serotonin system has been regarded for long time as a chief player among neurotransmitters involved in food intake regulation, so it is not surprising that it has been also under scrutiny in the search for molecular mechanism responsible of antipsychotics weight gain considering also that second-generation antipsychotics differ substantially in serotonin receptor occupancy compared to first generation ones. 5-HT is a potent satiety signal; administration of 5-HT to rodents decreases food intake [48]. Agonists at the 5-HT1A and 5-HT2C receptors have opposing effects on food intake: 5-HT1A agonists increase food intake [49] and 5-HT2C agonists decrease it as well [50]. 5-HT2C antagonists have been shown to increase food intake [51] and also attenuate the decrease in food intake which is produced by 5-HT2C agonists [52–54] or sibutramine, a 5-HT and noradrenaline reuptake inhibitor [55]. Knockout of the 5-HT2C receptor in mice can result in obesity and increased feeding [56]. Thus, the 5HT2C, 5HT3, and 5HT1A are the receptors with the most elevated probability to be involved in the induction of weight gain based on their physiological characteristics. The most studied receptor is 5-HT2C. What is surprising, however, is that the correlation between some of these receptors and weight gain is all but straightforward when different new generations of antipsychotics are compared to each other. Ziprasidone for example is an antagonist at the 5HT2C receptor, but there are no demonstrated clinical evidence for its weight-gain liability. Different findings suggest that serotonin also regulate NPY, one of the most abundant neuropeptides in the brain with high levels in several brain areas [57]. The 5-HT1B/2C agonist mCPP produced decreases in food intake, maybe through the reduction in NPY levels in the paraventricular nucleus (PVN) of the hypothalamus [58]. This is an important area of the brain which is involved in food intake and body-weight regulation, and it is rich in 5-HT2C [59, 60]. Furthermore, there is evidence suggesting an interaction between 5-HT2C and leptin, a circulating hormone that is released by adipocytes in response to increased fat deposition to regulate body weight and that acts through receptors mapping in hypothalamus [61]. 5-HT2C antagonists attenuate the reduction in food intake produced by leptin [61]. There are a body of evidences that 5-HT2C receptor and NPY are also involved in antipsychotics-induced weight gain. Chronic administration of clozapine produced an increase in NPY-immunoreactive cell density in the rat arcuate nucleus [62] that could, at least in part, be due to antagonism of the 5-HT2C receptor resulting in disinhibition of the NPY neurons. 

That the 5-HT2C receptor is directly involved in antipsychotics-induced weight gain is also demonstrated by pharmacogenetic observation such as the findings of Yuan et al. [63] that have identified several haplotypes of the promoter region of the 5-HT2C receptor gene which are associated with obesity and diabetes. Reynolds et al., in two different study [64, 65], have found that one 5-HT2C promoter polymorphism, −759*C/T*, is involved in developing weight gain during antipsychotics medication. In the first study, analyzed subjects (Chinese patients at first treatment) carrying the −759*T *allele had substantially lower weight gain following 10 weeks of treatment. In the second study, they have found that this association remains after 9 months of treatment in a group of first episode Caucasian patients. In the same study, the protective −759*T *allele was associated with higher concentrations of leptin.

## 4. Dopamine Signaling

Based on the pharmacodynamic profile of antipsychotics, dopamine has been considered the crucial target responsible for the efficacy of this class of compounds, and this may be true at least partially also for the new generation of antipsychotics since no compound devoid of D2R antagonism has been proved to be clinically effective in the treatment of schizophrenia [66]. Several lines of evidence also indicate that dopamine is involved in feeding behavior. (1) dorsal striatum and ventral striatum, both heavily innervated by dopamine, are related to the regulation of the caloric and rewarding effect of food, respectively [67]; (2) dopamine release is increased in certain areas of the brain, such as the accumbens, by palatable and sweet foods [68]; (3) dopamine may be critical in reward or in predicting the reward linked to food with high palatability [69]; (4) a reduced dopamine D2R availability measured with positron emission tomography and 11C Raclopride correlates with obesity in humans [67]. Moreover, the absence of dopamine production in the knockout mouse, which does not express tyrosine hydroxylase, does not inhibit motor capabilities to eat but causes an inability to initiate feeding; this ability can be restored by gene delivery of tyrosine hydroxylase into the striatum [70]. Dopamine release seems to have a site-specific action in food-intake regulation, such as in the nucleus accumbens, and it is associated with the reinforcement effect in feeding [71]. In the hypothalamus, dopamine release is associated with the duration of meal consumption, which is a factor in determining the feeding pattern. Hence, dopamine is required to initiate each meal and is thus associated with meal number and duration. Increased dopamine in the VMN, the area of neuroendocrine and autonomic regulation of metabolism, accompanies food intake. Medial hypothalamic lesions produce both hyperphagia and morbid obesity, suggesting that VMN dopamine is involved in regulating both food intake and the bodyweight setpoint [72–74]. 

The important contribution of dopamine to the regulation of food intake and feeding pattern has been shown in the medial hypothalamus by the study of both pre- and postsynaptic dopaminergic systems, as measured by in vivo microdialysis in experimental paradigms involving Zucker obese rat and its lean counterpart. Dopamine release is rapidly but transiently increased in the VMN during spontaneous eating in lean and obese rats, and this increase is more pronounced in obese rats, which consume larger meals [75]. When rats are deprived of food for 24 h, food intake brings about an immediate decrease of dopamine release to 65% of pre-eating levels in both lean and obese rats. These two findings show that release of dopamine is associated with both short-term (individual meals) and long-term (hunger) regulation of food intake. These data also suggest that during obesity, the presynaptic dopaminergic system adequately responds to the change of feeding status [73–75]. Dopamine acts on its receptors postsynaptically to modulate the activity of target neurons by stimulation or inhibition of adenylyl cyclase activity by D1 and D5 or D2 to D4 subtypes of receptors, respectively. The finding that hypothalamic neurons express both subtypes of dopaminergic receptors provides evidence that dopamine may activate or inhibit its target neurons based on the (1) function of the subtype and (2) level of the dopaminergic receptor expressed for a given metabolic condition under normal or pathologic conditions. D1 receptor, which normally is not abundantly expressed in the hypothalamus, was found highly expressed in the VMN of obese rats whereas D2 receptor was detected in the lean rat but at an undetectable level in the VMN of the obese Zucker rat [76]. The different levels of dopaminergic receptor expression in the two phenotypes may be related to the relatively low concentration of ligand (dopamine) in obese as opposed to lean rats, or it may directly depend on altered leptin signaling in postsynaptic neurons [76]. It is pointed out by different preclinical and clinical evidence that even with different degree, all clinically available antipsychotics have a certain degree of D2R occupancy. Beyond the clinical efficacy and extrapiramidal movements liability, the dopamine system has been investigated to much less extent for its implication in food regulation intake and weight gain during antipsychotic treatment. However, it should not be surprising that drugs that are with different degrees all antagonists of dopamine receptors may disrupt the normal physiology of dopamine-related feeding behavior, even if the precise mechanism by which dopamine could be primary affected is still elusive. On the other side, if dopamine is really involved in antipsychotics-induced weight gain, this possibility should be critically taken into account considered that dopamine depletion, at least in animals, is responsible of reducing food intake and that among antipsychotics with liability for weight gain, there are compounds with low affinity for dopamine D2 receptor. With regard to dopamine receptor involvement in antipsychotics-induced weight gain, it should be important to explore what extent the different dopamine receptors may contribute to feeding behavior and how antipsychotics with different D1-like and D2-like profile may differentially impact the body weight during the treatment. The putative role of dopamine system in the mechanism responsible for the weight gain is particularly puzzling, and the introduction in the pharmacotherapy of schizophrenia of antipsychotics with reported dopamine partial agonism does not help to clarify the issue. In 2005, it has been demonstrated that the administration of olanzapine (1–10 mg/kg p.o.) or aripiprazole (2.0–8.0 mg/kg p.o.) resulted in rapid and robust weight gain in rat taking the drugs compared to control animals whereas the liability for inducing prolactin elevation was substantially different, with olanzapine inducing a significantly increase in prolactin compared to control animals and aripirazole-treated animals [77]. The complexity of a “realistic” animal model of antipsychotics-induced weight gain is underscored by a series of experiments in which olanzapine and aripiprazole are compared. In a first experiment, the authors validated a pharmacologic isomorphism of olanzapine induced weight gain, by oral administration per 14 days, of olanzapine 5.0 mg/kg, twice per day, per os, in female Wistar rats [77]. As previously demonstrated with 8 mg/kg per 14 days olanzapine-induced a significant weight gain compared to control rats (vehicle treated) [78]. The weight gain was positively correlated with food intake, mirroring the increase in appetite in humans [79]; however, considering that weight gain can be detected starting from day 3 whereas food intake increase is detectable only from day 6, other factors should be taken into account in order to fully explain the mechanism of weight gain. In the second experiment, the different liability for inducing weight again and metabolic changes by antipsychotics with different receptor profile was challenged. The effect on weight gain of olanzapine compared to aripiprazole, a dopamine-partial agonist, was also studied in two different strains of rats: Wistar and Sprague Dawley. The utilization of two strains was introduced to gain information and evaluate the potential contribution of genetic background on antipsychotics-induced weight gain. Finally, in order to investigate the effect of housing conditions on food intake and weight gain, the authors compared the treatment with the two antipsychotics in rat singly and group housed [77]. Taking together all these findings suggest that DRD2 is a possible candidate gene for antipsychotics-induced weight gain. In 209 nondiabetic hypertensive and 174 gender-matched normotensive Chinese subjects, the DRD2 TaqI polymorphism was associated with obesity and hypertension [80]. 

Another gene belonging to dopamine pattern studied as a candidate gene for association study to obesity is the dopamine transporter gene. In a study, it was founded that the 10/10 genotype of the dopamine transporter gene has a 5.16 times higher likelihood of obesity in African-American smokers compared to the 9/9 or 9/10 genotype, while there was no association in non-Hispanic Whites [81].

## 5. Adrenergic Signaling

The role of the adrenergic system in the putative mechanism of antipsychotics-induced weight gain is elusive, even if a growing number of evidence indicate that both alpha and beta-adrenergic receptors are key molecules in the regulation of metabolic expenditure. Based on findings in animal models of obesity, the sympathoadrenal system has commonly been assumed to have a determining role in obesity development through its influence on regulation of energy expenditure. An earlier hypothesis was that sympathetic under activity, by reducing thermogenesis, might lead to weight gain in obesity, with some experimental observations supporting this view. One example was provided by surgical lesioning of the ventromedial hypothalamus in rodents, in which obesity was due to increased appetite and also to lowered sympathetic activity and obesity [82]. In regard of weight gain and body-weight homeostasis, it is proposed that excess in food intake is sensed by the brain which responds by a reduction of caloric assumption and by an increase in energy expenditure. This latter is mediated by sympathetic nervous system through the stimulation of adrenergic receptors on thermogenically active target tissues [83]. Taken one by one, single attempts including ablation of sympathetic nerves, the generation of genetically altered mice that are unable to synthesize catecholamines, and gene knockouts of individual *β* ARs have not resulted in obesity [84, 85]. It is possible that functional redundancy between the three known *β* ARs can justify the lack of complications caused by loss of all adrenergic signaling. To prove this hypothesis, Bachman et al. have created mice that are knockout for all three known *β* ARs (*β*-less) [86, 87]. This triple knockout mice that have lacked the *β*1/*β*2/*β*3-adrenoceptors developed a progressive obesity at adulthood. An involvement of the adrenergic system in antipsychotics-induced weight gain is suggested by pharmacogenetic investigations that have shown an association between the *β*2 adrenergic receptor gene polymorphisms and obesity. Although not undisputed [88, 89], two meta-analyses of a number of human studies have found the Trp64Arg polymorphism in the beta3-adrenergic receptor gene to be associated with obesity [90–92]. Several recent studies confirmed this association [93–96]. A pilot study of 73 subjects with schizophrenia [97] also found a trend (*P* = .10) between clozapine-related weight gain and the Trp64Arg beta3-adrenergic receptor gene, with the presence of the less frequent arginine allele predicting weight gain. The affinity of antipsychotic agents to the alpha1 adrenergic receptor also suggests that this gene may be a candidate for pharmacogenetic investigation of antipsychotics-induced weight gain. In addition, alpha 1-noradrenergic binding was inversely proportional to body weight gain [98], and in animal model of diet-induced obesity it was founded that lower levels of alpha 1-noradrenergic receptor in the hypothalamic ventromedial nucleus were expressed only in those rats that became obese. In a study involving 60 treatment-refractory patients with schizophrenia, Basile et al. [97] found a trend (*P* = .22) towards reduced clozapine-induced weight gain in individuals homozygous for the cysteine variant of the Arg347Cys polymorphism of the alpha 1-adrenergic receptor gene. With respect to drug-induced weight gain, no preclinical data are available on the effect of antipsychotics on central beta receptors. The two compounds with the highest liability for weight gain, clozapine and olanzapine, have no significant affinity for any of the beta receptors; however, it has been demonstrated that olanzapine-induced activation of prefrontal cortical neurons, analyzed by c-fos induction, is prevented by administration of the beta-receptor antagonist propanolol [99]. It has been hypothesized that olanzapine acting on presynaptic alpha 1 receptors may increase the release of norepinephrine in the prefrontal cortex and in turn activate adrenergic beta receptors [99]. It will be interesting to show a similar c-fos induction and propanolol prevention in neurons of CNS regions related to food intake or energy expenditure.

## 6. Hystamine Signaling

Hystamine is probably the neurotransmitter for which there are more available data on an involvement in food intake regulation. Histaminergic neurons are exclusively found in the tuberomammillary nucleus in the posterior hypothalamus but act through postsynaptic H1-receptors on almost all areas of the brain involved in energy homeostasis. Moreover, several reports have suggested that histamine (HA) is involved in the regulation of arousal state, locomotor activity, cardiovascular control, water intake, food intake, and memory formation on antihistaminic receptorial activity of antipsychotics. The propensity of antipsychotic agents to induce weight gain is addressed by different experimental on affinity for H1-receptor system. Many authors have founded that affinity for H1 receptors closely correlates with antipsychotics-induced weight gain [100]. Consistent with these data, other authors have demonstrated that histamine H1-receptor antagonism promotes feeding in rodents and that H1 knockout mice are prone to weight gain [101, 102]. Moreover, in this animal model is abolished clozapine augmentation of AMP kinase that is potentially stimulated in the hypothalamus by orexigenic antipsychotics and is involved in regulating food intake [103]. Also, leptin-induced food intake appears suppresses in H1 knockout mice. This data suggest that that hypothalamic histamine is a modulator of leptin activity [104, 105]. Furthermore, histamine release in CNS seems to be involved in lipolysis [106] and glycolysis [107] during energy-deficient states in animal studies. There are several novel H1-receptor polymorphisms that are been identified, including Leu449Ser [108], −17 C/T, −974 C/T, −1023 A/G, −1536-G/C [109], and Glu349Asp [110], but there is only one pharmacogenomic study focused on antipsychotics-induced weight gain that shows no association in 88 schizophrenic patients between the Glu349Asp variant of the HR-1 gene and weight gain after 4 months of treatment with clozapine [110], but the use of treatment-refractory schizophrenic as samples probably may have diminished the power of association study. Despite the relevance of these neurobiological factors, it is notable that different authors have pointed out that the drugs with high H1 receptor antagonism can lead to weight gain also for their sedative effects with consequent reduced mobility.

## 7. Cholinergic Signaling

Some evidence have suggested that the blockade of mACh exerted by different antipsychotics may lead to weight gain trough anticholinergic-induced dry mouth that has as a consequence an increase in the ingestion of high-calorie beverages. One study showed a significant reduction in muscarinic M2 receptor density in the dorsal vagal complex DVC and a significant increase in weight gain in rats after treatment with olanzapine but not with haloperidol [111]. 

The DVC comprises the dorsal motor nucleus of the vagus (DMV), nucleus of the tractus solitarius (NTS), and the area postrema (AP). The NTS is the primary site for innervation by vagal afferents from the gut, whilst the DMV is the site for primary efferents to the gut [112]. The DVC is also a target for the inhibitory effect of leptin on food intake [113]. There is evidence that the DVC is also involved in detecting and responding to hunger and satiety signals. This is an indirect involvement of cholinergic transmission in weight gain induced by antipsychotics mechanism. Nevertheless, neither remarkable weight gain nor diabetes mellitus has reported the use of typical anticholinergic drugs, such as scopolamine. This finding reinforce the data of Matsui-Sakata et al. [114] that in a meta-analysis pointed out that the affinities to mACh receptors of the antipsychotics were very low and correlates with H1 receptor occupancy. As a consequence, mACh receptor occupancy is likely to be a confounding factor in analysis of the cause of antipsychpotics-induced weight gain.

## 8. Cannabinoid Signaling

The endocannabinoid system consists of cannabinoid receptors, endocannabinoids, and a set of endocannabinoid synthesising and degrading enzymes. Early roles for the endocannabinoid system were ascribed based on the location of the endocannabinoid receptors. In the early 1990s, two cannabinoid receptors, belonging to the family of G-protein coupled receptors, had been cloned cannabinoid receptor type 1 (CB1) and cannabinoid receptor type 2 (CB2), and the two major endogenous cannabinoids (the so-called endocannabinoids) were identified as arachidonoyl ethanolamine (anandamide, AEA) and 2-arachidonoyl glycerol (2-AG). Therefore, the abundance of CB1 receptors in the brain suggested a role as an important modulator of neuronal functions, and the fact that CB2 receptors are mainly expressed in the immune system suggested an immune modulatory role. In addition to other functions, endocannabinoids have been shown to induce a dose-dependent orexigenic effect and thus mimic the hyperphagic effect produced by THC [115]. More importantly, it has been demonstrated not only that changes in endocannabinoids production occur with respect to the feeding state of the animal, for example, increasing between meals, reaching a critical level in order to trigger the motivation to feed, and then falling rapidly following access to food, but that these changes are localised to specific areas of the brain directly involved in modulating feeding behaviour, such as the nucleus accumbens [115]. Some authors hypothesize that the endocannabinoid system may display an incentive action to increase the incentive value of the food regardless of the quality of the macronutrients [116, 117]. Others speculate about the orosensory rewarding properties of endocannabinoids [118]. In other words, endocannabinoids may stimulate a preference for highly palatable food. Interestingly, CB1 receptors are colocalised with dopamine D1 and D2 receptors in the rat limbic forebrain and crosstalk between the endocannabinoid and the dopaminergic system in the control of reward for tasty food has recently been documented using dopamine D1 receptor antagonists which reduce the orexigenic stimulus induced by THC [119]. Several findings in rodents point to a pivotal role of the hypothalamus as the integrating centre for the anabolic signals induced by endocannabinoids [120]. In that regard, recent evidence suggests that endocannabinoids act within various hypothalamic nuclei to influence other neurons that produce peptides that influence food intake. In lateral hypothalamus, one finds the following.

1. It has been demonstrated that AEA increases and AM251 (a CB1 antagonist) decreases depolarization-induced neuropeptide Y (NPY) release in hypothalamic rat explants, suggesting that NPY may contribute to the orexigenic action of endocannabinoids—possibly via modulation of an intermediate factor involved in NPY release [121].
2. CB1 receptor stimulation strongly augments the orexin-A-stimulated intracellular pathway, and this effect can be blocked by the CB1 receptor antagonist rimonabant, suggesting a positive orexigenic role for CB1 in this neural population [122].
3. In models of obesity such as *ob*/*ob* and *db*/*db* mice, characterised by an impairment of leptinergic signalling, it has been shown an increase of (pathological) levels of endocannabinoids at the hypothalamus. A single intravenous (i.v.) injection of leptin in these animals was able to reduce the overproduction of endocannabinoids [123].
4. Recently, it has been also characterised the interactions among endocannabinoids, leptin, and melanocortin-concentrating hormone (MCH) within the limbic system: MCH neurons are inhibited by GABAergic inputs from the limbic system and endocannabinoids act to reduce GABA release, and thus may stimulate the excitability of MCH neurons, leading to an increase in food intake [123, 124].
5. Endocannabinoids also interact with a-melanocyte-stimulating hormone (MSH) and cocaine- and amphetamine-related transcript(CART). This is a neuropeptide highly expressed in the hypothalamus and nucleus accumbens. Hypothalamic CART has been associated with food intake and body weight control [125].

The hypothalamic paraventricular nucleus (PVN) may represent another site within the brain where endocannabinoids interact with orexigenic and anorexigenic neurons. Here, it has been clearly demonstrated that endocannabinoids released from parvocellular neurons act at presynaptic CB1 receptors to decrease glutamatergic transmission onto CRH-releasing neurons, resulting in an inhibition of CRH release. Recent findings suggest that the orexigenic crosstalk between endocannabinoids and ghrelin signalling may represent a novel target for the pharmacological treatment of obesity [126]. In order to investigate the role of cannabinoid system in antipsychotics-induced weight gain, Weston-Green et al. have investigated whether different antipsychotic drugs (olanzapine, haloperidol, aripiprazole, or vehicle) alter cannabinoid receptor-binding density in the DVC in female Sprague-Dawley rats treated in both acute (1 week) and chronic (12 weeks) paradigms [127]. Using quantitative autoradiographic methods, they have found that acute olanzapine induced a significant 39% decrease in cannabinoid receptor binding compared to controls, whilst short-term aripiprazole and haloperidol had no significant effect. In addition, chronic olanzapine treatment induced a significant 46% decrease in cannabinoid receptor binding compared to controls, and aripiprazole slightly decreased cannabinoid receptor binding (12%), whilst haloperidol had no effect [127]. These results coincide with the clinical setting in which patients treated with olanzapine exhibit higher weight gain than aripiprazole [128] and haloperidol [129]. However, due to the low abundance of CB2 receptors in the DVC, it is probable that the findings observed in this study was mostly to the CB1 receptor. This study supports a role for brainstem cannabinoid receptors in the mechanisms of antipsychotic-induced weight gain. In contrast with Weston-green et al., Theisen et al. [130], in a recent work, using radioligand-binding assays to compare the binding affinities of clozapine, olanzapine, and haloperidol for candidate receptors potentially involved in AP-induced weight gain, have not found significant binding rate to cannabinoid receptor 1.

## 9. Insulin, Leptine, and Other Neuropeptide Signaling

In 1979, it was demonstrated in nonhuman primates that insulin infused into the CNS caused a significant decline in food intake and body weight. Woods et al. [131] proposed that insulin served as an “adiposity signal” that links the behavior of feeding with size of adipose stores [131]. In the mid-1990s, it was identified leptin as another candidate hormone working in adiposity signal [132] and has been well characterized as a regulator of energy homeostasis. The presence of insulin in the CNS was reported in 1979 [133]. Although negligible quantities of insulin can be synthesized locally, many studies established that the predominant amount of insulin in the CNS can be accounted for by receptor-mediated transport into the CNS by blood [134]. Receptors for both insulin and leptin are widely expressed throughout the CNS, but the major target of these two hormons is the medial hypothalamus [134]. Although the leptin receptor is present as different splice-variant isoforms in the CNS, the form OBRb has the major role in its metabolic action [135]. The obese *db*/*db* mouse and Zucker *fa*/*fa* rat represent naturally occurring “knockouts” of the leptin receptor that have helped to validate the importance of CNS leptin action in energy homeostasis. Leptin and insulin have multiple effects on energy homeostasis through activation of key hypothalamic nuclei and peptides to regulate energy balance such as neuropeptide-Y (NPY), POMC and its product *α*-melanocyte-stimulating hormone (*α*-MSH), and the melanocortin antagonist, AgRP [136, 137]. POMC and AgRP are selectively expressed in neurons of the ARC nucleus colocalized with receptors for insulin and leptin, and different data suggested that leptin and insulin act on ARC to increase melanocortin and decrease AgRP to regulate food intake and energy balance [138]. Recent evidence suggest that there are other key players in modulation of food intake influenced by insulin and leptin in a direct or indirect way. Among these, are notable Orexin A and melanin concentrating hormone (MCH), both with orexigenic function. Additionally, Orexin A may be an important factor in the effects of drugs of abuse and for the rewarding effects of food [137]. The orexins (hypocretins) are a family of hypothalamic neuropeptides that are selectively expressed in neurons of the classical “feeding area” of the lateral hypothalamus and perifornical area (LH/PFA) [139, 140]. The orexins (orexin A and orexin B) arise from the precursor peptide prepro-orexin by proteolytic processing. 

Orexins bind and activate two closely related G-protein-coupled receptors, orexin 1 receptor (OX1R) and orexin 2 receptor (OX2R) [139]. Despite the small number of orexin cells, orexin axons are distributed throughout the brain [141, 142]. The orexins are potent regulators of feeding and other metabolic processes and of arousal [143, 144]; in addition, intracerebral orexin administration increases food intake [145]. In a study of Fadel et al. (2002) [146], the authors have demonstrated in animal experimental paradigm that *fos *expression was induced in orexin neurons by APDs that cause significant weight gain but not by those APDs with low weight-gain liability. However, amphetamine also activated orexin neurons, but clozapine markedly increased *fos *expression in orexin neurons that innervate the PFC whereas amphetamine had a weak effect. This findings suggest that it is likely that changes in the activity of afferents or hormonal signals to these orexin neurons determine the differential response. The identity of these afferents is unknown, but may include the PFC and nucleus accumbens, regions that receive dopaminergic projections from the A10 cell group and project to the LH/PFA [147, 148]. Moreover, the receptors that are targeted by APDs that induce weight gain may be present on orexin neurons or on afferents to the orexin neurons, such as neurons of the PFC and play an important role. Alternatively, peripherally derived signals may be critical. Several data suggested the presence of specific CNS circuitry which is either directly or indirectly connected with hypothalamic circuitry to modulate feeding behavior. Components of this circuitry could contribute also to complex behaviors like food seeking and food intake and include portions of the cerebral cortex, hippocampus, amygdala, and the striatonigral pathway, which is implicated in transposing motivational aspects of stimuli into motor responses, as well as hedonic evaluation of the stimulus and associative learning. The major neurotransmitter pathways associated with motivation and hedonics are mesolimbic dopamine (DA) and certain CNS opioid pathways. In terms of neural connectivity, the hypothalamus is linked to the motivational circuitry of the CNS, and numerous mono- and multisynaptic pathways between different components of the limbic circuitry and the hypothalamus have been identified [149]. Food intake can be driven by energy demands, that is, “homeostatic” feeding and by the palatability or pleasure associated with eating a preferred food, “non-homeostatic” feeding [150]. The behavior associated with non-homeostatic feeding is in part regulated by the mesolimbic dopamine system. Berridge et al. have identified nucleus accumbens (NAc) dopamine projections, that receive projection of DA neurons from VTA as central to “wanting” and has been implicated in the motivating, rewarding, reinforcing, and incentive salience properties of natural stimuli such as food and water as well as drugs of abuse [151]. The neural mechanisms of food reward are believed to be similar, if not identical, to drug rewards. 

At least, Ghrelin is a newly discovered appetite-stimulating peptide that has a role in the regulation of feeding behaviour. It is primarily secreted by the stomach and duodenum, and it has been implicated in both mealtime hunger and the long-term regulation of body weight. Ghrelin is currently recognized as the main endogenous ligand for growth hormone secretagogue receptors. The levels of circulating ghrelin are increased under conditions of starvation and in anorexia nervosa, but decreased under conditions of feeding and in obesity. Ghrelin and leptin may have opposite actions in the regulation of body weight. The link between atypical antipsychotic treatment and elevated serum ghrelin levels is not clear so far, but a dysregulation of the central feedback mechanism can be hypothesized. Murashita et al. in an interesting work have suggested that olanzapine may directly act on the secretion of ghrelin and induce appetite, resulting in weight gain [152]. 

## 10. Animal Model of Weight Gain after Antipsychotics Administration

A critical approach to the search for a molecular mechanism of antipsychotics weight gain should start recognizing that the results from animal models have been not consistent and that the paradigms may not fulfill the hypothesis: quite a paradox for compounds whose action on CNS can be modeled with relatively sufficient approximation. On the other side, a reliable animal model of antipsychotics-induced weight gain should be particularly helpful considering that this side effect cannot be completely predicted on the basis of the receptor-binding profile of an antipsychotic agent. The importance of animal models is underlined also by a recent consensus development on antipsychotics drugs, obesity, and diabetes, and it is recommended in order to determine whether the above disorders are due to a central peripheral mechanisms. Several caveats needs to be taken into account analyzing animal models: (1) the route of administration, (2) the day-time of administration, (3) the duration of the treatment, (4) the dose-equivalent to human dose, and (5) species difference, in some of these methodological issues may be relevant for the modeling of increased food intake whereas others for the reproduction of the metabolic dysregulation that me be responsible of weight gain in patients taking atypical antipsychotics. 

The route of administration is usually i.p. or i.m. injection, and only few studies have used oral administration and the cleavage method. Chronic injection and cavage may represent a stressful condition that can interfere with food-intake regulation and should be carefully controlled. The day time of drug administration may be relevant considering that rodents have an inverse day-night rhythm compared to human, and this method of treatment may have consequences on food intake. The antipsychotic dose administered is chosen mainly based on the dopamine D2 occupancy and on the behavioral effects of the compound. The effect of certain antipsychotics on metabolism and weight gain in different species may be different from the dose responsible for the CNS effects. The duration of the treatment may be a crucial issue too; the mean duration of chronic animal studies is about three–four weeks, the time frame in which is believed the antipsychotic action begins to appear. However, the metabolic effects in human appear clearly after 10 weeks of treatment and are usually reported within this frame time. Finally, few studies have been conducted in a “head-to-head” antipsychotics-administration paradigm that includes more than two antipsychotics. The results of animal studies conducted have been in some cases surprising. Both olanzapine (2.5–5 mg/kg/day) and haloperidol (0.08–0.31 mg/kg/day) administered by cavage for three weeks to male and female Wistar rats induced a significant weight gain in female rats only, failing to reproduce the clinical situation where olanzapine has been shown to increase weight gain significantly more than haloperidol in both genders. Moreover, the low but not the high dose of olanzapine caused a significant increase in food intake in female rats [153]. If antipsychotics influence food intake and this effect may in turn be responsible for the weight gain, how the meals pattern is changed? In other words, what is modified the feeding rate or the meal size or both? The meals pattern of free feeding in rats has been studied by Lee and Clifton who have investigated meal behavior in Lister hooded rats after the i.p administration of haloperidol (0.05–0.2 mg/kg), clozapine (1–10 mg/kg), olanzapine (0.3–3.0 mg/kg), and analysis of feeding rate and meal size in the interval of time between 2 h and 48 h after the drug injection [154]. Haloperidol and clozapine, but not olanzapine, produced a transitory increase in food intake in the 2 h following drug administration. A rapid compensatory response to the initial period of hyperphagia was indicated by the unchanged total amount of food intake after six hours. Haloperidol produced a substantial enhancement of meal sizes at intermediate doses whereas higher doses were related to a decrease in meal size Clozapine and olanzapine showed no tendency to enhance meal sizes, and the highest doses of olanzapine and haloperidol decreased meal sizes. This study indicates that feeding rates and meal sizes at least in this experimental paradigm are under control of different mechanisms [154]. Are animal models useful in dissecting the effect of low and high antipsychotics dose? Although risperidone has been associated with less weight gain than clozapine, risperidone treatment leads to a greater increase in the body mass of patients than conventional treatment with an antipsychotic such as haloperidol. 

A study from Ota et al. (2002) revealed an intriguing biphasic response to low and high doses of risperidone with respect of weight gain [155]. In this study, rats were injected twice a day with risperidone 0.005 mg/kg, 0.05 mg/kg, and 0.5 mg/kg for 21 days, the body weight was measured, and the expression of leptin and uncoupling protein 1 (UCP-1) were quantitated with Real-Time PCR. Leptin is mainly expressed in the white adipose tissue (WAT); UCP-1 is a protein promoting energy expenditure in the Brown Adipose Tissue (BAT). Risperidone 0.005 mg/kg increased food intake and the rate of body weight gain, as well as *leptin *gene expression in WAT. Risperidone 0.5 mg/kg caused a reduction in body weight and an increase in UCP-1 expression. It appears clear that a critical issue in developing a realistic animal model of weight-gain induction after chronic treatment with antipsychotics should rely on a “quasi-naturalistic” modality of drug administration. As already mentioned, the injection of the drug allows a precise delivery of the daily dosage; however, it may be stressful for the animal and have a negative impact on the behavioral outcome; on the other side, administering the drug with the food is also biased by the need to house the animal in a single cage that may mimic an isolation paradigm. A new method of oral antipsychotics administration in individual rats has recently been proposed [156]. The animals (male Sprague Dowley rats) were trained to drink 2 ml of 5% sucrose solution from a disposable syringe on a daily basis for 1 week or until each animal had taken the 2 ml of fluid from the syringe on three consecutive days. After three week of training, all the animals were adapted to drink the solution. At this point, the treatment was initiated according to the experimental protocol: the animal were divided in four groups, haloperidol (2 mg/kg), clozapine (20 kg/mg), diazepam (0.5 mg/kg), or vehicle solution (control treatment) were administered; all the drugs were dissolved in a 5% sucrose solution and were administered for 4 or 7 weeks. The results of the behavioral testing (vacuous chewing movuments, grooming disengage test, chocolate disengage test, and motivational test) was consistent with each drug treatment. After four weeks, only the animals treated with diazepam group showed a significant weight gain compared to controls and to all other treatments; however, after seven weeks, all the animals treated with an active compound showed a similar increase in body weight significantly different from the control group. The animals that were treated for four weeks and then withdrawn from the treatment were not significantly different in body weight from the rats of the control group. This study indicates that this novel method of drug administration may be considered reliable and specifically suitable for studying antipsychotics-induced weight gain, avoiding the potential bias due to the cavage or drug injection [156].

## 11. Weight Gain and Insulin Function in Animal Model

In addition to weight gain, patients treated with atypical compounds display various metabolic disturbances including increased serum triglycerides and total cholesterol as well as decreased high-density lipoprotein cholesterol [157]. Several other studies have reported anomalous responses to the oral glucose tolerance test and elevated fasting insulin levels after treatment consistent with insulin resistance [158]. Initially, these alterations in glucose regulation and other metabolic parameters were seen as secondary to the weight gain linked to atypical antipsychotic use. However, rapid induction of hyperglycemia, sometimes accompanied by ketoacidosis, has been reported in patients on clozapine and olanzapine without weight gain challenged this notion [159]. This line of evidence suggests a direct, drug-related effect that is independent of changes resulting from weight gain. Despite the burgeoning clinical literature on the possibility that atypical antipsychotics may induce diabetes, few studies have tackled in an animal model, in vivo, the relationship between second-generation APS, weight gain, and pancreatic function. The effect on weight gain, abdominal fat, insulin sensitivity, pre- and post- administration of olanzapine (dose target.15 mg/day) o or risperidone (dose target 5 mg/day) has been investigated in mongrel dog compared to a “neutral” treatment (gel capsules) [160]. Abdominal MRI, euglicemic hyperisnsulinemic clamp (measuring haepatic and insulin sensitivity) and a stepwise beta-cell stimulation (monitoring pancreatic beta-cell response) were performed. This study not only reveals differential effects of two atypical antipsychotics on adiposity and hepatic insulin resistance but also shows for the first time a substantial effect of one antipsychotic to impair pancreatic *β*-cell function. Animals treated with olanzapine exhibited greater (+5.9%) weight gain than which (+3.9%) were observed with RIS. The authors also found that observed body-weight changes are not explained solely by alterations in food intake, but it reflects treatment effects on both caloric intake and energy expenditure. MRI analyses revealed that dogs treated with olanzapine developed substantial adiposity that greatly exceeded observed changes in control animals. At variance, ripseridone caused more modest increases. In regard of glucose metabolism, in this study, the authors have pointed out that dogs treated with olanzapine exhibited severe hepatic insulin resistance (reduced above 75%). This differs with the more modest declines with risperidone or placebo. While olanzapine-induced hepatic insulin resistance may reflect a primary hepatic defect, other sites of action are also plausible. This study revealed a dramatic impairment in *β*-cell compensation during olanzapine treatment. Both drugs induced similar whole-body insulin resistance, yet only riperidone responded with compensatory upregulation of *β*-cell sensitivity. Since insulin secretion can be neurally regulated, one possibility is that negative effects of olanzapine on *β*-cell compensation may be mediated by its known central actions as a dopamine antagonist. Moreover, it is unknown if a similar dopamine-mediated neuropancreatic axis exists for pancreatic endocrine secretion. Since studies have suggested that weight gain and therapeutic response resulting from clozapine treatment may be correlated [161], Zhao et al. in their interesting work have examined behavioral and biochemical effects of clozapine [162]. 

They have measured startle response and prepulse inhibition of the startle reflex in untreated mice and 68-day clozapine-treated mice. They have found that clozapine treatment significantly reduced the startle response but did not potentiate prepulse inhibition. In the same work, they have also treated mice with clozapine (2 mg/kg/day) for 3 days, 25 days, and 68 days. These treatments developed a sustained insulin insensitivity, which resulted in impaired plasma glucose clearance as measured by intraperitoneal glucose-tolerance test without significant weight gain. Indeed, impaired plasma glucose clearance was observed at day 3 and persisted for the entire 68-day period of clozapine treatment. This suggests that clozapine was effective not only in behavioral changes as measured by the startle response magnitude but also in systemic metabolic alterations. If the mechanistic relationship between weight gain and diabetes induced by antipsychotics has been investigated only in few animal studies, the mechanism by which second generation-antipsychotics may disrupt the normal metabolic function and cause glucose disturbance has been investigated in *in vitro *experiments mainly addressing the effects of antipsychotics on glucose transport and glucose sensing [163, 164]. Trying to make sense of the preclinical and *in vitro* data on the relationship between putative mechanisms responsible of weight gain and diabetes in schizophrenics patients treated with second-generation antipsychotics, one should take into account the important observation that diabetes may be a disorder that is related with pathophysiology of schizophrenia and as underlined recently by Citrome. He has assessed that the presence of a diagnosis of schizophrenia in itself is a risk factor, prompting the Canadian Diabetes Association to recommend routine screening for diabetes for all who have that disorder. Lifetime risk of developing diabetes may in fact be quite high for certain groups, as estimated by the Centers for Disease Control and Prevention, where the estimated lifetime risk of developing diabetes for individuals born in 2000 appears to be 32.8% for males and 38.5% for females, with the highest estimated lifetime risk for diabetes being among Hispanics (males 45.4%, females 52.5%) [165]. Moreover, in its recent meta-analysis, the same author did not not demonstrate a significantly increased risk of diabetes during treatment with second-generation antipsychotics compared with first-generation drugs [165]. This finding suggests that probably basic studies have overscored the importance of the disorders in glucose metabolism induced by second-generation antipsychotics and probably underlie that insulin resistance and schizophrenia have a common substrate.

## 12. Conclusions

Weight gain due to antipsychotic medication is associated with significant physical and psychological morbidity. Individuals with schizophrenia treated with antipsychotics have concerns about weight gain and weight management, like the general population, and express a desire to be more active. Driven by the public health impact of obesity, substantial progress has recently been made in the basic understanding of the regulation of appetite and body weight, including the identification of genetic polymorphisms and other markers of obesity risk. Newer studies are focusing on elucidating the fundamental mechanisms of weight gain through pharmacogenomic approaches and evaluating changes in energy homeostasis, metabolism, and energy intake and expenditure during antipsychotic treatment. As psychiatric practice moves into an era of personalized medicine, the ability to identify risk markers, besides those related to demographic characteristics or the drug itself, will not only help clinicians to screen out patients at high risk for weight gain and metabolic changes, but potentially allow earlier access to higher-risk medications for patients who do not possess vulnerability markers. Medications such as olanzapine and clozapine carry significant metabolic burdens, but are effective treatments for some patients who do not respond to other antipsychotics. The elucidation of mechanisms by which antipsychotic medications impact metabolic parameters remains important for quantification of patient risk, to inform the frequency and targets of metabolic monitoring during antipsychotic therapy, and to permit the development of novel agents without these limitations.
